# Employees’ Weekend Activities and Psychological Well-Being via Job Stress: A Moderated Mediation Role of Recovery Experience

**DOI:** 10.3390/ijerph17051642

**Published:** 2020-03-03

**Authors:** Jae-Geum Jeong, Seung-Wan Kang, Suk Bong Choi

**Affiliations:** 1 College of Global Business, Korea University, 2511 Sejong-ro, Sejong City 30019, Korea; 2 College of Business, Gachon University, Seongnam 13120, Korea

**Keywords:** job stress, psychological well-being, recovery experiences, weekend activities, moderated mediation effect, hierarchical multiple regression

## Abstract

An employee’s off-work activities are known to contribute positively to recovering their energy levels depleted by daily work. Despite this view and understanding, the effect of employees’ weekend activities on their psychological well-being has not attracted sufficient research interest. Therefore, the purpose of this paper is to analyze the relationship between employees’ weekend activities and their psychological well-being, and the mediating role of job stress in the above relationship. We also investigated the moderating role of the recovery experiences in the relationship between employees’ weekend activities and job stress. Furthermore, we examined the moderated mediating effect of recovery experiences on the relationship among employees’ weekend activity, job stress, and psychological well-being. The survey data was obtained from 294 employees working in 15 manufacturing companies in South Korea. The participants were 71.1% men and 28.9% women, 49.7% were university graduates, followed by 26.2% college graduates, 12.6% high school graduates, 10.2% post-graduates, and 1.4% Ph.D. holders. In terms of age composition, 50% participants were in their thirties, followed by 19.7% in their forties. The empirical analysis revealed that weekend activities are positively associated with employees’ psychological well-being. Moreover, job stress was found to mediate the relationship between weekend activities and psychological well-being. We also found that the recovery experiences positively moderated the relationship between weekend activities and job stress. Further, the study revealed that the higher the level of recovery experience, the greater the effect of weekend activities on psychological well-being affected by job stress. The paper also discusses the theoretical and practical implications of the study.

## 1. Introduction

The work-related aspects of employees in today’s business environment have become very complex, as employees increasingly need to cope with dynamically changing workplace situations [1]. Work-related activities make up the largest proportion of everyday life of employees. These activities are generally accompanied by a sense of duty, and a heavy demand is imposed on employees to execute the roles assigned to them, which results in an increase in stress, thus negatively affecting their mental and physical state [2,3,4]. However, people generally engage in work-related activities in pursuit of a happy life, which is the ultimate goal of any individual’s life. Happy employees are more creative in their work and are more committed to their organizations, which contributes to reducing employee turnover and absenteeism [5,6,7,8,9]. It is therefore important to relieve the stress that accumulates during the work week through an appropriate stress management system. The negative effects of stress are not transient but recur constantly, increasingly depleting the employees’ resources and adversely affecting their physical and psychological health [10,11,12,13]. To prevent this, stress should be managed in a timely and appropriate manner.

As a means of coping with job stress, weekend activities and recovery experiences have been attracting public interest, which has fueled a surge of related research [14,15,16,17,18]. However, previous studies on job stress and psychological well-being have addressed the issue from an organizational perspective [19,20,21]. Recently, it was reported that employees’ weekend activities help recover the energy resource they expend at work [22,23]. The activities were also found to enhance their feeling of personal well-being by improving their mental and physical states and were thus seen as being positively associated with regular work activities [24,25]. However, despite the growing interest in the association between weekend activities and job stress, there is still a lack of research on the effect of weekend activities on the psychological well-being of employees, and the effectiveness of weekend activities is still open to debate [18,25]. Therefore, the question arises as to whether weekend activities have anti-stress effects and improve psychological well-being of all employees. In particular, it was observed that psychological detachment, relaxation, recovery of resources, and psychological stability are not proportional to the quantity of weekend activities but vary according to individual characteristics and the degree of personally perceived recovery experiences [26]. In this respect, this study seeks to broaden the understanding in four areas [16,17,18].

First, we examine whether weekend activities have a positive effect on employees’ psychological well-being. Weekend activities, as proposed by Sonnentag [18], include low-effort social, physical, and intellectual activities. Employees tend to engage in complex activities over the weekend rather than concentrating on a specific activity. Accordingly, there will be individual differences in the perception of psychological well-being from the overall activities undertaken during the weekend.

Second, we aim to investigate whether employees’ weekend activities can positively contribute to reducing job stress. Job stress is a conflict that inevitably arises in the relationship between individual capability and job environment [27]. Greenberg and Baron [28] defined job stress as the emotional and physical patterns that arise in response to an environment that is perceived by each individual as a threat to his/her goal when completing their job. The main focus of this study is on the negative stress associated with role overload, role ambiguity, and role conflict that employees experience while executing their job activities. Such stress adds to the psychological burden, impacts self-confidence with regard to the job content and performance, and intensifies feelings of anxiety. In other words, job stress is a state of depleted resources due to work activities, with both body and mind fatigued. This stress gradually builds up and accumulates over time [29]. It is therefore essential to recover from stress using appropriate methods, and weekend activities are considered effective for stress reduction.

Third, previous studies [24,30,31] have demonstrated that stress and psychological well-being are closely related. This is ascribable to the positive effect of weekend activities on the recovery of depleted mental and physical resources and reduction of stress accumulated during the week, which in turn leads to psychological well-being. Therefore, this paper analyzes the mediating role of job stress in the relationship between employees’ weekend activities and their psychological well-being.

Fourth, this paper examines the moderating effect of workers’ recovery experiences on the relationship between employees’ weekend activities and job stress. Recovery experiences during off-work time include those experiences that restore the fatigued mental and physical state to the baseline state [32] and replenish the depleted resources to an optimal state [33]. Although weekend activities generally have a positive effect on the psychological well-being, the extent to which employees actually feel and perceive the recovery as a result of weekend activities varies depending on the intensity of the recovery experiences. Thus, this study also investigates the moderated mediating role of recovery experiences in the relationship between weekend activities and the psychological well-being affected by job stress.

**Figure 1:**
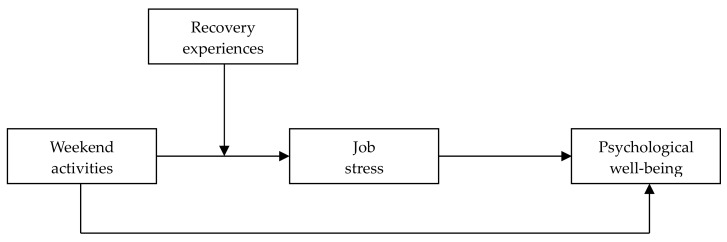
Hypothesized research model.

Figure 1 shows our analytical model. By addressing these issues, this study seeks to not only address the effects of employee’s off-work activities, but also to provide insights on how managers can improve employee’s psychological wellbeing in the workplace.

## 2. Theoretical Background and Hypotheses

### 2.1. Weekend Activities and Psychological Well-Being

Weekend activities comprise a wide variety of activities undertaken over the weekend after a week’s work, including general leisure activities, rest, work, and household chores. They include different forms of general leisure activities. Leisure activities are personal activities that are free of job stress and pressure, undertaken during off-work time, including the weekend, in order to rest the body and mind and replenish lost energy by engaging in various cultural and social activities. Loosely [34] defined leisure activities as activities undertaken in pursuit of self-development or voluntary participation in social activities for recreation or relaxation away from the workplace or home duties and responsibilities. Atchley and Barusch [35] defined leisure activities in a broader sense as all activities undertaken by one’s own choice rather than a social phenomenon.

Orthner [36] classified leisure activities into individual, parallel, and joint leisure activities. Individual leisure activities are activities that can be carried out alone without contacting or building relationships with others, such as meditation or training. Parallel leisure activities are activities which involve little interaction with others, such as watching TV or movies and listening to music. Further, joint leisure activities are those involving physical contact or relationship building with others, including various sports activities. However, weekend activities include several more activities in addition to the leisure activities mentioned above. Sonnentag [18], in a study on recovery activities, classified weekend activities into five categories: (1) Work-related activities (finishing tasks, preparation for upcoming work week, personal care); (2) household and child-care activities (cooking, dish-washing, shopping, and child-care); (3) low-effort activities (watching TV, taking a bath); (4) social activities (chatting, taking a phone call); (5) physical activities (sports, cycling, dancing). This classification helps clearly distinguish the degree of effort or energy required for carrying out different types of weekend activities that have been theorized to have differential effects on workers’ resources [18].

Weekend activities are not mandatory but voluntary activities. However, dealing with work-related tasks over the weekend is an additional stressor and adversely affects employees’ physical and psychological well-being. In contrast, low-effort activities will likely lead to mental and physical rest and recovery of resources since they are passive activities and may not require expending valuable physical and mental resources [37]. Moreover, empirical studies have demonstrated a positive relationship between social and physical activities and personal health and well-being [38]. In fact, Sonnentag [18] attributed the potential contribution of social activities in resource recovery to two mechanisms. First, social activities provide an opportunity for social support, which has a huge effect on restoring resources [39]. Second, people pursuing leisure activities that involve social contact experience more positive effects than those who pursue activities devoid of social contact [40] since social activities do not require resources necessary for work. Furthermore, in the effort-recovery model [41] and conservation of resources theory [42], the recovery process was proposed to play an important role in individual health and well-being. Everybody perceives accumulated mental and physical fatigue or stress as a negative factor, and there arises a potential need to resolve it [42], which is expressed through rest or the pursuit of leisure activities. In particular, given the time constraints on the activities at the end of a workday, weekends are usually used for pursuing activities that help in stress relief and recovery from the week’s exertion. Such activities can be interpreted as the manifestation of the desire to relieve stress and fatigue accumulated from job activities during the week. Given that weekend activities are voluntary activities, they include a high level of self-control and are isolated from rules or external influences. Moreover, employees can pursue activities as per their will. Thus, weekend activities are proposed to have a positive effect on workers’ psychological well-being, as appropriate weekend activities can contribute to improving and restoring psychological and physical health.

Ryff [43] proposed six key dimensions of psychological well-being: self-acceptance, personal growth, purpose in life, environmental mastery, positive relations with others, and autonomy. Self-acceptance refers to a positive attitude towards self by acknowledging and accepting multiple aspects of self and feeling positive about past life. The dimension of positive relations with others refers to the ability to build harmonious interpersonal relationships through affection, attention, intimacy, trust, and well-being in interactions with others. Next, autonomy means self-determination and independence, the ability to think and act on one’s own accord, and strong self-control. Further, environmental mastery is the ability to manage the environment through familiarity with the surroundings and to make effective use of the environment or steer it towards a suitable context. Purpose in life is having goals and meaning in life, bestowing meaning on present and past life, and holding beliefs. Lastly, personal growth refers to the aspiration of continued self-development, to self-perception as a growing and expanding being, being open to new experiences, being aware of one’s own potential, and expecting to improve over time. Therefore, weekend activities are autonomous and self-directed activities for self-realization and expansion of goals and meaning in life to achieve a sense of well-being and are expected to be highly correlated with positive relations with others. Thus, we hypothesized the following.

**Hypothesis** **1.** *Employees’ weekend activities have a positive effect on their psychological well-being.*

### 2.2. Mediating the Effect of Job Stress

Job stress is expected to have a mediating effect on the relationship between weekend activities and psychological well-being due to the following reasons. First, work-related activities during a week include various factors such as increasing task demands, role conflict, and pressure [44], and the job stress accumulated over years in a workplace environment can have a negative effect on employees’ mental and physical health [3,4,45,46]. Weekend activities contribute to stress relief by replenishing the depleted mental and physical resources [47,48]. When the potential risk of one’s situation is perceived, job stress will be low if sufficient physical and mental resources are available; if not, the stress level will be high. In other words, if the resources can be replenished, negative perception can be avoided even in the face of a negative situation [49]. From the viewpoint of the conservation of resources theory [50,51], however, absence of sufficient resources would increase negative perception and stress, which would further deplete resources. This situation entails efforts to compensate for the insufficient resources with alternative resources, whereby weekend activities can be used as an appropriate means of alternative resources, which may reduce job stress. Second, marked by subjective individual autonomy, weekend activities are aimed at pursuing personal development, growth, and expansion, as well as relieving job stress and replenishing resources. They enhance psychological well-being through overall satisfaction with life and self-realization, helping in maintaining positive relations with others, emphasizing the meaning of life, strengthening the goals for a better life, and helping achieve efficient management of life in all aspects [43]. Therefore, weekend activities have a positive effect on the employees’ psychological well-being. Third, job stress is an important factor which decides the extent to which weekend activities have a positive effect on psychological well-being, i.e., the results can vary depending on the degree of job stress [24,25]. Thus, an employee experiencing a high level of stress for lack of weekend activities would have lower psychological well-being than an employee with a low level of stress [52]. Many studies have demonstrated that workplace stress has a negative effect on individual health and well-being [3,53,54,55,56]. In summary, weekend activities efficiently reduce job stress, which, in turn, increase psychological well-being. However, while weekend activities can help relieve a certain level of job stress and enhance psychological well-being through mental detachment from work, they cannot help to relieve a high level of job stress, which leads to a decrease in psychological well-being. Based on this claim and empirical research, it is expected that the level of job stress mediates the relationship between weekend activities and psychological well-being. Thus, we hypothesized the following.

**Hypothesis** **2.** *Job stress mediates the relationship between weekend activities and psychological well-being.*

### 2.3. Moderating Effect of Recovery Experiences

This study proposes that recovery experiences moderate the relationship between weekend activities and job stress. The conservation of resources theory [42] hypothesizes that people strive to obtain, retain, and protect resources. For example, employees seek to recover depleted resources through weekend activities as part of such an effort. Recovery is the process of replenishing used-up resources and restoring the previous state [33,57]. The extent to which such recovery takes place varies depending on the type of weekend activities and individual personalities. Recovery experiences can be assumed to be the response to and result of weekend activities [32,48]. Therefore, understanding recovery experiences can help gauge the relationship between weekend activities and job stress. Recovery experiences arise from the perception of the process or result of weekend activities. If weekend activities cannot elicit feelings or awareness of recovery, the level of perception is low, as is the level of recovery. Pursuing low-effort activities or social activities as weekend activities lead to positive recovery experiences, as these activities need fewer resources [58]. Moderate physical activities are also expected to help relieve stress by reducing mental and physical tension, and thus enhancing the level of recovery experiences. In fact, the higher the level of recovery experiences, the greater the positive effect of weekend activities. Considering the effect of recovery experiences on intensifying the positive impact of weekend activities and thus reducing job stress, we proposed the following hypothesis:

**Hypothesis** **3.** *Recovery experiences moderate the relationship between weekend activities and job stress. In other words, weekend activities help reduce job stress more effectively when recovery experiences are perceived more intensively.*

### 2.4. Integrated Model: Moderated Mediation Effect

In light of the hypotheses proposed in previous studies, the moderation pattern hypothesized above refers to a moderated mediation where the mediating variable works as a function of the third variable [59]. More specifically, the more intense the recovery experiences, the greater the positive effect of weekend activities on reducing job stress. In this relationship, a decrease in job stress—the mediating factor in the relationship between weekend activities and psychological well-being—intensifies the positive effect of weekend activities on psychological well-being. In contrast, in the case of weak recovery experiences, the positive effect of weekend activities decreases, failing to efficiently reduce job stress and its negative effect on psychological well-being, thus diminishing the perceived psychological well-being. Therefore, it is expected that recovery experiences moderate the effect of psychological well-being on weekend activities based on the mediation of job stress. Following this rationale, the below hypothesis was proposed:

**Hypothesis** **4.** *The relationship between weekend activities and psychological well-being through the mediation of job stress will vary depending on the level of recovery experiences. In other words, the higher the level of recovery experiences, the greater the effect of weekend activities on psychological well-being mediated by job stress.*

## 3. Method

### 3.1. Sample and Procedure

In this research, a survey was conducted, which mainly included the managers and employees from 15 manufacturing companies in South Korea where overtime work at night is a frequent occurrence. The reasons for selecting these employees for our survey were as follows: (1) We can more precisely grasp the recognized condition of weekend activities and psychological well-being from Korean employees as they generally experience greater stress than workers from other developed countries because of more frequent night-time work; thus, (2) they are an appropriate group to study the effect of stress in such a situation. Questionnaires were distributed to the employees after acquiring permission from the chief manager who was explained the purpose of the survey. By providing enough information on the survey to their boss, the major concern of the participants that their responses could be seen by the company and their boss were alleviated, leading to more accurate responses. The participants were requested to answer the weekend activity part of the questionnaire based on their weekends from the previous month and not merely on the previous weekend. A total of 320 copies of the questionnaire were collected out of 390 copies (82.09%), and finally data from 294 questionnaires were analyzed after eliminating incomplete copies. The demographic characteristics of the survey were as follows. The participants included 71.1% men and 28.9% women. Further, with regard to age, 50% participants were in their thirties, followed by 19.7% in their forties, 18.7% in their twenties, 10.2% in their fifties, and 1.4% in their sixties. Of the participants, 49.7% were university graduates, followed by 26.2% college graduates, 12.6% high school graduates, 10.2% post-graduates, and 1.4% Ph.D. holders. With regard to their position, the participants included 52.7% staff members, 18.7% administrative managers, 12.7% section chiefs, and 15.6% participants had a rank above team leader.

### 3.2. Measures

#### 3.2.1. Weekends Activities

We measured the employees’ weekends activities using Sonnentag’s [18] 16-item scale. These included low-effort activities (watching TV; reading magazines, newspapers, books; listening to music), social activities (religious gatherings, other gatherings, services, and social activities), physical activities (sports and leisure activities: Mountaineering, camping, soccer, volleyball), intellectual activities (related to educational learning: School attendance, hobby learning, job-related self-learning). The questionnaire included one question for each activity, i.e., a total of four activity-based questions. An example of a question is, “I do a lot of low-effort activities (watching TV; reading magazines, newspapers, and books; listening to music).” Responses were divided on a scale of 1 to 5 (1 = very much, and 5 = never). Employees cannot have time to spend on other activities if they spend time on certain activities. Internal consistency checks were excluded as the activities in the category were not correlated [60].

#### 3.2.2. Recovery Experience

Recovery experience was measured using a 12-item scale developed by Sonnentag and Fritz [32], which covers the three dimensions of recovery experience: Psychological separation, relaxation experiences, and control experiences. Each dimension contained four items, with a combined total of 12 items. Sample items for each of the dimensions included “I forgot about work,” “I indulged in relaxing activities,” and “I sought intellectual challenges,” respectively. The response format was based a five-point scale, ranging from 1 (strongly disagree) to 5 (strongly agree). Cronbach’s α of the summative scale was 0.93.

#### 3.2.3. Job Stress

We used a four-item scale developed by Keller [61] to assess the level of the employees’ job stress. Sample items included, “I experience stress from my job” and “I never feel pressured in my job.” The response format was based on a five-point scale, ranging from 1 (strongly disagree) to 5 (strongly agree). Cronbach’s alpha was 0.88 in this study.

#### 3.2.4. Psychological Well-Being

To measure the psychological well-being of employees, 19 items were used utilizing six components (Self-Acceptance, Positive Relations with Others, Autonomy, Environmental Mastery, Purpose in Life, and Personal Growth) developed by Ryff [43]. Sample items included “Possesses a positive attitude towards self,” “Has warm, satisfying, trusting relationships with others,” “Has goals in life and a sense of directedness,” and “Has a feeling of continued development.” The response format was based on a five-point scale, ranging from 1 (strongly disagree) to 5 (strongly agree). Cronbach’s alpha was 0.91 in this study.

## 4. Results

The descriptive statistics and zero-order correlations of the study’s variables were displayed in Table 1. As our survey data was collected from 15 organizations, we evaluated whether the responses differed across organizations by using ANOVA(analysis of variance) test. The result showed no significant organizational membership effect on our model.

### 4.1. Confirmatory Factor Analysis

As seen in Table 2, confirmatory factor analysis (CFA) was performed to test construct validity of four study variables (i.e., weekend activities, recovery experiences, job stress, and psychological well-being). The hypothesized four-factor model indicated satisfactory fit to the data (χ^2^ = 1020.78, df = 645, CFI = 0.939, TLI = 0.934, RMSEA = 0.045). Further, we set three alterative models and conducted the Chi square comparison test with the hypothesized model. We found that all three alternative models differed from statistically from the hypothesized model, and their fitness to data were relatively worse than the hypothesized model.

### 4.2. Hypothesis Tests

The results of hierarchical regression analyses are presented in Table 3. Hypothesis 1, weekend activities were positively associated to psychological well-being (*β* = 0.28, *p* < 0.001), was tested after controlling for gender, age, education level, position, and job assignment characteristics with the weekend activities in Model 5. Following the test procedure developed by Baron and Kenny [62], we tested the mediational role of job stress in the weekend activities–psychological well-being relationship (Hypothesis 2). First, by testing Hypothesis 1, we had already confirmed the positive effect of weekend activities on psychological well-being. Second, in Model 2, from the participants’ evaluation of weekend activities, it was found that weekend activities had a positive effect on them and reduced their work stress. Therefore, weekend activities reduce job stress (*β* = −0.36, *p* < 0.001). Finally, in Model 6, job stress was positively associated to psychological well-being (*β* = −0.19, *p* < 0.01), indicating significant additional variance in psychological well-being. In addition, we also tested the meditating role of recovery experiences in the relationship between weekend activities and psychological well-being. We found that recovery experiences positively mediated the above relationship (see Appendix A).

**Figure 2:**
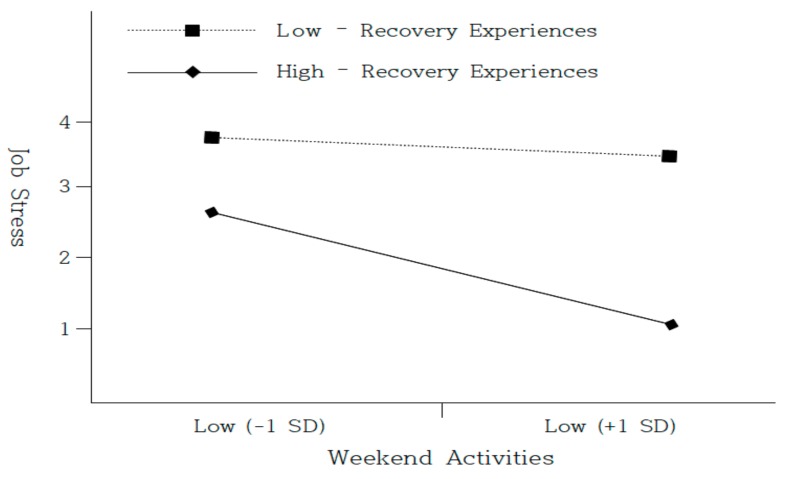
Moderating effect of recovery experiences on the relationship between weekend activities and job stress.

Further, it was found that the effect of weekend activities on psychological well-being was strong (*β* = 0.24, *p* < 0.001), indicating partial mediation. To prove this result even more, the bootstrap method suggested by Preacher and Hayes [63] was conducted. The results indicated a significant indirect effect (indirect effect = 0.05, SE(standard error) = 0.04, 95% CI [0.13, 0.31]). Thus, Hypothesis 2 was proven. Regarding the moderating role of job stress, the interaction term of weekend activities and recovery experiences significantly predicted job stress (*β* = −0.42, *p* < 0.05; Δ*R*^2^ = 0.28) in Model 3. Furthermore, we illustrated an interpretation of the interaction pattern in Figure 2 [64].

As shown in Figure 2, the positive relationship between weekend activities and job stress was stronger when recovery experiences were high (simple slope = −0.34, t = −3.90, *p* < 0.001) than when it was low (simple slope = 0.04, t = −3.18, *p* < 0.01). Thus, Hypothesis 3 was supported. We continued to test Hypothesis 4, investigating whether the indirect effect of weekend activities on psychological well-being via job stress was moderated by recovery experiences. To assess the conditional indirect effect, Hayes’ [65] PROCESS program was applied. The indirect effect of weekend activities on psychological well-being via job stress was estimated at high (+1 SD) and low levels (−1 SD) of recovery experiences. The results confirmed that the indirect effect was significant for high recovery experiences (conditional indirect effect = 0.03, SE = 0.01, 95 % CI [0.01, 0.06]) but was not significant for low recovery experiences (conditional indirect effect = 0.01, SE = 0.01, 95 % CI [−0.00, 0.04]). Therefore, Hypothesis 4 was proven.

## 5. Discussion

### 5.1. Theoretical Contributions

This study investigated the individual benefits of employees’ weekend activities. Specifically, the study examined the mediating role of job stress and the moderating role of recovery experiences in the relationship between employees’ weekend activities and psychological well-being. The results of this study have the following theoretical and practical implications: First, our results revealed that employees’ weekend activities are an essential factor for enhancing their psychological well-being. Previous studies mostly focused on the employees’ recovery activity and process during the weekend and effects of weekend activities on job performance [25,47,48]. However, the effect of weekend activities on individual psychological status, such as psychological well-being, has rarely been studied. Moreover, weekend activities have a mixed outcome that yield both positive and negative effects on the recovery of individual mental resources and job performance [25,47]. This study extends the existing literature by empirically demonstrating the positive and essential benefits of weekend activities on employees’ psychological well-being.

Second, our study found the key mediating mechanism to explain how weekend activities promoted employees’ psychological well-being. Several studies have examined how weekend or leisure activities influenced employees’ attitude toward their job and life such as job stress, life satisfaction, and recovery experiences [17,22,33,47,48]; however, the relationships among weekend activities, job stress, and psychological well-being have been rarely investigated. In particular, by identifying the substantial mediating role of job stress, this study provides useful insights to understand the key mechanism of how weekend activities facilitate psychological well-being of employees.

Third, this study suggests that recovery experiences are an important moderator in the relationship between weekend activities and job stress. Our study contributes to the existing study by identifying recovery experiences as a vital situational variable that can improve the effect of weekend activities. Much previous research has simply explained the degree of recovery as an outcome measure of weekend activities [17,23]. This study, however, expanded on the previous research by identifying the differences of weekend activities’ effect for job stress depending on the level of recovery experiences. Furthermore, our findings also show that the level of recovery experiences is also an important conditional variable for the indirect relationship among weekend activities, job stress, and psychological well-being. Consequently, our findings provided a more integrated picture regarding the roles of weekend activities in relation to employees’ psychological well-being.

### 5.2. Managerial Implications

The practical implications of this study are as follows. First, given the empirical findings of positive effects of weekend activities on reducing job stress and ultimately improving psychological well-being of employees, we suggest that organizations in Korea should pay attention to provide their employees with the work conditions that guarantee desirable weekend activities. Managers also need to recognize the importance of weekend activities for improving employees’ personal quality of life and job performance and should facilitate the organizational climate and job schedule so that employees can have a proper weekend activity.

Second, considering the negative effect of job stress on the relationship between weekend activities and psychological well-being, managers need to make continuous efforts to ensure adequate stress management for employees. At the same time, an organization should develop various supporting schemes for employees’ weekend activities based on their opinions, needs, and social information, as this is an effective non-work factor to reduce job stress [47,48,52,58], which ultimately influences organizational performance.

Finally, our findings also suggest that managers should recognize the individual’s level of recovery experiences to maximize the positive effects of weekend activities on reduction of job stress and enhancing of psychological well-being. Therefore, it may be a worthwhile effort for managers to often communicate with employees who have had high levels of recovery experiences and recovery abilities through weekend activities, and disseminate and apply the desirable recovery experiences when they consult the employees who are struggling with job stress and bad quality of psychological well-being.

### 5.3. Limitations and Future Research

This section discusses the limitations of this study and future research directions. First, the data used for this study were limited to a specific country and industry, with mostly supervisor-level employees, posing the problem of generalizability, which will have to be addressed in future research by expanding the scope of data collection. Second, the study data were collected using a cross-sectional design, which involves difficulties in identifying the accurate state of the respondent. Given the time-dependent nature of weekend activities or job stress and their relationship with psychological well-being, longitudinal data collection is necessary for future research. Third, while our findings are valuable in that they synthesize the effects of various weekend activities, the analysis in this study was focused on non-work-related activities. However, we did not investigate work-related weekend activities, such as job preparation and wrapping up weekday jobs. We also did not analyze all possible non-work-related types of weekend activities, such as stressor-type activities, including household and child-care activities [18]. These types of weekend activities may dynamically interact with the activities we have examined in this study. Therefore, it would be worth comparing the effects across these different types of work-related and unrelated weekend activities in the future research.

Finally, there may be various psychological factors connected with the improvement of psychological well-being resulting from weekend activities. This study presupposed an autonomous nature of weekend activities. In case of married employees, however, timing and methodology of weekend activities may be subject to personal and familial adjustment due to different desires. Weekend activities converging the needs of the family irrespective of personal needs will likely increase stress because it requires personal resources. Furthermore, perception of well-being may vary depending on the psychological reference point for psychological well-being. For example, if the psychological reference point for psychological well-being is the family, the employees prioritize their weekend activities as per the family’s preference even if these activities have a negative effect on their own mental and physical state. With this in mind, there is a need to conduct further research to develop complex research models by considering various contingency variables.

## 6. Conclusions

Our study investigated the effect of employees’ weekend activities on their psychological well-being. Our findings reinforce that job stress plays a mediating role in the effect of weekend activities on promoting psychological well-being. We also examined the role of recovery experiences in facilitating a better condition for the above relationships. The study highlights the positive benefits of weekend activities, and the role of recovery experiences in the relationship between weekend activities and job stress, ultimately strengthening the effect of weekend activities on psychological well-being. Despite the limitations, the study provides invaluable insights that can help managers understand the mechanisms and conditions that explain how employees’ weekend activities increase their psychological well-being in the workplace in Korea.

## Figures and Tables

**Table 1:** Means, standard deviations, correlations, and reliabilities.

Variables		M	SD	1	2	3	4	5	6	7	8	9
1.	Gender	1.29	0.45	-								
2.	Age	2.25	0.92	−0.87	-							
3.	Education	2.61	0.88	0.21 **	−0.23 **	-						
4.	Position	1.91	1.13	−0.08	0.44 **	0.07	-					
5.	Job characteristics	2.34	1.77	−0.00	0.11	−0.09	−0.02	-				
6.	Weekend activities	2.44	0.67	0.09	0.03	−0.04	−0.07	−0.09	-			
7.	Recovery experiences	2.48	0.91	−0.00	−0.02	−0.05	−0.19 **	−0.13 *	0.33 **	(0.93)		
8.	Job stress	3.01	0.66	−0.03	0.00	−0.00	0.10	0.12 *	−0.38 **	−0.062 **	(0.88)	
9.	Psychological well-being	3.50	0.53	0.15 **	0.08	0.10	0.02	−0.05	0.31 **	0.19 **	−0.29 **	(0.91)

Notes: *n* = 294. * *p* < 0.05; ** *p* < 0.01 (two-tailed test). Values in parentheses along the diagonal are Cronbach’s alphas.

**Table 2:** Model fit statistics for measurement models.

Model	χ^2^ (df)	CFI	TLI	RMSEA	Δχ^2^ (Δdf) ^a^
Hypothesized four-factor model (WA, RE, JS, PW)	1020.78 (645)	0.939	0.934	0.045	
3-factor model (WA & RE merged, JS, PW)	1605.85 (662)	0.847	0.838	0.070	585.07 (17) ***
2-factor model (WA & RE merged, JS & PW)	2722.44 (664)	0.667	0.647	0.103	1701.66 (19) ***
1-factor model	3835.68 (665)	0.487	0.457	0.128	2814.90 (20) ***

Notes: WA: Weekend activities, RE Recovery experiences, JS: Job stress, PW: Psychological well-being, CFI: Comparative fit index, TLI: Tucker–Lewis index, RMSEA: Root mean square error of approximation; ^a^ Chi square difference for each model reflects its deviation from the four-factor model. *** *p* < 0.001.

**Table 3:** Hierarchical multiple regression for job stress and psychological well-being.

Variables	Job Stress			Psychological Well-Being		
	Model 1	Model 2	Model 3	Model 4	Model 5	Model 6
Gender	−0.02	0.01	−0.01	0.14 *	0.11	0.11
Age	−0.09	−0.05	0.02	0.14 *	0.11	0.10
Education	−0.01	−0.02	−0.04	0.10	0.12	0.11
Position	0.14 *	0.10	0.01	−0.04	0.01	0.01
Job characteristics	0.13 *	0.09	0.01	−0.06	−0.01	−0.00
Weekend activities		−0.36 ***	0.03		0.28 ***	0.24 ***
Recovery experiences			−0.28 *		0.11	0.00
Job stress						−0.19 **
Weekend activities × Recovery experiences			−0.42 *			
*R* ^2^	0.03	0.16 ***	0.44 ***	0.04 *	0.14 ***	0.17 ***
Δ*R*^2^		0.13 ***	0.28 ***		0.10 ***	0.03 ***

Notes: *n* = 294. Standardized coefficients are reported; * *p* < 0.05; ** *p* < 0.01; *** *p* < 0.001.

## Appendix A

**Table A1:** Hierarchical multiple regression for recovery experiences and psychological well-being.

Variables	Recovery Experiences		Psychological Well-Being		
	Model 1	Model 2	Model 3	Model 4	Model 5
Gender	−0.02	−0.08·	0.07 *	0.09	0.10
Age	0.08	0.06	0.04 *	0.10	0.09
Education	−0.03	−0.01	0.03	0.11	0.11
Position	−0.18 ***	−0.16 ***	0.03	0.00	0.02
Job characteristics	−0.07 **	−0.05 *	0.01	−0.03	−0.01
Weekend activities		0.41 ***		0.36 ***	0.32 ***
Recovery experiences					0.13 *
*R* ^2^	0.06	0.15 ***	0.04 *	0.17 ***	0.18 ***
Δ*R*^2^		0.09 ***		0.13 ***	0.01 ***

Notes: *n* = 294. Standardized coefficients are reported. * *p* < 0.05; ** *p* < 0.01; *** *p* < 0.001.
